# Estimates of adherence to treatment of vivax malaria

**DOI:** 10.1186/1475-2875-13-321

**Published:** 2014-08-15

**Authors:** Eduardo D Almeida, Luiz Carlos S Rodrigues, José Luiz F Vieira

**Affiliations:** Laboratório de Toxicologia da Universidade Federal do Pará, Rua Augusto Correa, 01- Campus Universitário do Guamá, Belém-Pará, Zip code: 66075-740 Brazil

**Keywords:** Adherence, Malaria, Compliance, Reliability therapeutic failure, Efficacy

## Abstract

**Background:**

The relation between therapeutic failure and non-adherence to treatment of malaria has been clearly established. Several measures have been used to estimate adherence to *Plasmodium vivax* therapy, but few protocols have been validated to ensure reliability of the estimates of adherence. The objective of this study was to validate a five-item-reported-questionnaire derived from original Morisky four-item scale to estimate adherence to *P. vivax* malaria therapy.

**Methods:**

A five-item-reported questionnaire was applied to patients after treatment of *P. vivax* malaria, considering behaviours regarding to forgetfulness, carelessness as to time of administration, cessation or discontinuation of use and replication of dose. Data were collected in dichotomous and Likert scales. Reliability was assessed by Cronbach’s alpha and by the contribution of each item to total. The concurrent validation was done with pill count and concordance between measures of adherence by coefficient of Kappa. Sensitivity, specificity and accuracy were also determined.

**Results:**

A total of 135 patients were enrolled in the study. Adherence ranged from 63.8 to 72.7% in both psychometric measures and pill count. The responses on the Likert scale showed higher proportion of non-adherence behaviour, greater variance and concordance with pill count, as well as more sensitive to characterize the behaviour of self-medication. The internal consistency of questionnaire was moderate. Significant correlations were seen with items regarding the forgiveness or careless in taking pills in all scales. The agreement between psychometric measures and pill count was considered satisfactory. The non-adherence to malaria therapy in an endemic area of Amazon basin was 33.3%.

**Conclusion:**

The five-item-reported questionnaire with responses on Likert scale is a feasible option for reliable estimation of adherence to malaria therapy in endemic areas.

## Background

Malaria by *Plasmodium vivax* is a major health problem in Amazon basin, with approximately 200,000 cases reported annually [1]. The standard treatment is based on the association of chloroquine plus primaquine according to the recommendations of World Health Organization [2]. However, treatment failure has been reported throughout the world, including in Brazilian Amazon basin, where it has been estimated at 10-15% [3–5]. Generally, it has been associated both to resistance to anti-malarials drugs and to decreased concentrations of drugs in the bloodstream caused by incorrect dosage, poor drug quality, drug interactions, variations in pharmacokinetics parameters of drugs, and poor patient adherence in respect of either dose or duration of treatment [6].

The treatment adherence ensures the complete recovery of patients and prevents the emergence of parasite resistance [7]. There are several determining factors in adherence to malaria therapy, such as perception of disease, the complexity of the schedule, the quality of prescription, or the patient’s clinical improvement [8]. The non-adherence to malaria therapy has been estimated at 2-40%. A study in Brazilian Amazon basin showed low adherence in 13.5% of patients [9].

Several measures have been used to estimate adherence, such as pill count, use of electronic monitoring devices, patient’s interview before and after treatment about dose, days and frequency, and measurement of the levels of drugs or a metabolite in the bloodstream [10–14]. Methods of self-reporting by the patient have been widely used, and when properly structured and applied may provide a reasonable accurate estimate of adherence [15]. Morisky *et al.* [16] proposed a four-item-self-reported structured questionnaire on a sample of hypertensive patients to assess medication adherence, considering that drugs errors or omission could occur in any or all of several ways: forgetting, carelessness, stopping the drug when feeling better or starting the drug when feeling worse. Since then, several improvements were made to increase the reliability of Morisky questionnaire, as the addition of new items, changes in response scales and concurrent validation [17–22].

The direct relationship between therapeutic failure and non-adherence with treatment has been clearly established for several diseases, but few studies have evaluated the use of psychometric measures for estimating adherence in neglected diseases. The objective of this study was to validate a five-item-self-reported questionnaire derived from the original Morisky questionnaire, with the addition of an item regarding self-medication behaviour, to be used to estimate adherence to malaria therapy in endemic areas. Responses were compared between dichotomous and Likert Scales and pill count was used for concurrent validation.

## Methods

### Study area

The study was carried from September to October 2012 at the Basic Unit Health at Anajas-PA, a rural municipality at Marajo Island (0°59′21” S and 49°56′24” W), with an area of 6.921 Km^2^. The population was estimated at 26,547 inhabitants in 2013. The transmission of malaria is intense with some seasonal fluctuation. Anajas reported in the last years the highest numbers of cases of *P. vivax* in the state of Para.

### Sample size

The sample size was determined using an expected non-adherence proportion of 13%, with significance level 95% and precision level of 5%. Therefore, the study would require the participation of 135 patients with malaria by *P. vivax* [23].

### Patients and treatment

In this study were enrolled adults with slide-confirmed infection by *P. vivax*. Exclusion criteria were: patients with mixed malaria or with signs and symptoms of severe malaria (jaundice, renal or pulmonary impairment, severe anaemia, altered level of consciousness), parasite density over 5%, glucose-6-phospate dehydrogenase deficiency, and anti-malarials drugs treatment prior to inclusion in the study. Each subject received a multiple oral doses of chloroquine (10 mg/kg on Day 0 and 7.5 mg/kg on days 1 and 2) co administered with primaquine (0.50 mg/kg/d for seven days, as 13.2 mg primaquine phosphate tablets) [2]. This study received approval from the Institute Evandro Chagas Ethical Committee (CEP/IEC/SVS/MS-0028/2010). All subjects provided written consent before participation.

### Data collection

Data were collected by two trained research assistants to administer the structured five-item-self-reported questionnaire, the use of informed consent forms and the procedures of questioning the participants [24]. The original Morisky questionnaire was translated to Portuguese by naive English spoken. Patients were monitored daily in the community health center and those who reached the seventh day of the medication were visited at their homes to interview. The five-item-self-reported questionnaire was completed by counting any remaining pills, both of chloroquine and primaquine.

### Measurements

Adherence was estimated using a five-item-self-reported questionnaire derived from the four-item original of Morisky. The five yes/no questions were: Do you ever forget to take your pills? Are you ever careless in taking your pills? Do you ever miss taking your pills when you are feeling better? Do you ever miss taking any of your pills because you are feeling sick? Do you replicate the dose when you are feeling sick?

Initially, the patients were randomly allocated in two groups; Group 01 (n = 66), who answer questions in dichotomous scale (DS) as yes/no, in which a “no” answer received a score of 1, and a “yes” answer received a score of 0; and Group 02 (n = 69), who answer questions on a six point Likert scale (LS), as “all the time”, “nearly always”, “usually”, “sometimes”, “once a while” and “never” [25]. Then the Likert scale was dichotomized (LDS) in order to increase the sampling effort. The answers as “all the time”, “nearly always”, “usually” and “sometimes” were classified as yes (0) and “once a while” and “never” classified as no (1). Finally LDS and DS were grouped into an overall dichotomous scale (ODS), which was correlated with pill count. Levels of adherence to anti-malarial therapy in the LS were determined by the sum of the percentage of each item divided by the total of the item, and in the dichotomous scales by simply adding each item. The values below the median were used to allocate patients in the non-adherent group.

The reliability in each scale was assessed by internal consistency of questionnaire and by item-to-total, which was used to determine the contribution of each item to the overall reliability. Internal consistency was compared between original Morisky and five-item-self-reported questionnaires.

Pill count consists of a simply counting the number of dosage units that the patient has not taken by the scheduled appointment. The returned dosage units are counted and compared with the number of units received by the patient. Medication regimen adherence is calculated by subtracting the number of units returned from the number of units issued. The amount used is then divided by the expected amount and multiplied by 100 to determine the percentage of compliance. A full adherence was considered when patients taken all pills (100%). Patients taken above 70% were classified as “probably adherent” and below 70% as “non-adherent”. If all pills returned to the medical staff, the patients were considered as “certainly non adherent”. Then the patients probably adherent and with full adherence were classified as adherent, whereas the patients probably non-adherent and certainly non-adherent as non-adherent.

Concordance between psychometric measures and pill count was estimated by Kappa coefficient. Concurrent validation of psychometric measures was done with pill count followed by determination of sensitivity, specificity and accuracy.

### Statistical analysis

Data are presented as median and range. The Chi-square was used to compare categorical variables. Reliability was assessed by Cronbach’s alpha and by the contribution of each item-to-total, determined by Spearman’s Rank correlation [26]. Cronbach’s alpha also was used to determine the reliability of original Morisky and five-item-self-reported questionnaires. The concurrent validation between pill count and psychometric measures was done by Spearman’s Rank correlation. The coefficient of Kappa estimates the concordance between pill counts with psychometric measures [27]. Distribution of probabilities was used to determine the sensitivity, specificity and accuracy of questionnaire. The analysis was performed using SPSS 21.0 (SPSS Inc. Chicago Il). The significance level accepted was 5%.

## Results

A total of 135 patients were included in the study in age from 18–52 years (mean 36 years). Sixty percent of the patients were male (95%CI: 51.1-67.4). The descriptive analysis of psychometric measures is show in Table 1. Likert scale showed the highest variance.

**Table 1:** **Descriptive analysis of the total scales scores in psychometrics measures**

MMAS	Median	Range	Variance
Dichotomous (n = 66)	0.6	0.26-0.88	0.063
Likert (n = 69)	4.68	3.84-5.12	0.280
Likert dichotomized (n = 69)	0.54	0.22-0.71	0.048
Overall dichotomous (n = 135)	0.57	0.24-0.79	0.055

Adherence behaviour ranged from 63.8 to 72.7%. There were significant differences between adherent and non-adherent groups, but the estimate in each group were similar in all measures of adherence to treatment. The higher proportion of non-adherence was seen in LS (95% CI: 24.6-46.4) and the lower in DS (95%CI: 16.7-37.9) (Table 2).

**Table 2:** **Adherence and non-adherence to treatment estimated by psychometric measures and by pill count**

Measure of adherence to treatment	Non-adherence		Adherence		***p-values***
	(%)	95%CI	(%)	95%CI	
Dichotomous (n = 66)	*27.3*	(16.7-37.9)	*72.7*	(60.6-81.8)	<0.0001*
Likert (n = 69)	*36.2*	(24.6-46.4)	*63.8*	(52.2-72.5)	0.0078*
Likert dichotomized (n = 69)	*34.8*	(24.6-43.5)	*65.2*	(53.6-73.9)	0.0033*
Overall dichotomous (n = 135)	*31.1*	(23.0-37.8)	*68.9*	(61.5-74.8)	0.0002*
Pill count (n = 135)	*28.9*	(21.5-34.8)	*71.1*	(63.0-77.8)	<0.0001*

*Statistically significant.

Cronbach’s alpha values ranged from 0.609 to 0.672 and 0.508 to 0.717 in the original Morisky and in the five-item-self-reported questionnaires, respectively. The internal consistency was moderate (>0.5), with high values in LS in both questionnaires (Table 3).

**Table 3:** **Internal consistency of original Morisky and five-item-structured questionnaires**

Measure of adherence to treatment	Morisky	Five-item
Dichotomous (n = 66)	0.609	0.508
Likert (n = 69)	0.672	0.717
Likert dichotomized (n = 69)	0.630	0.657
Overall dichotomous (n = 135)	0.621	0.599

Cronbach’s alpha values.

The item-to-total correlation coefficient ranged from −0.07 to 0.59 (DS), 0.26 to 0.72 (LS), 0.20 to 0.70 (LDS) and 0.15 to 0.65 (ODS). Significant correlations were seen with items regarding the forgiveness or careless in taking pills (i-1 and i-2) in psychometric scales. A negative correlation was seen with the fifth-item in DS. Only the LS showed significant correlation after the introduction of the fifth item. Cronbach’s alpha values decreased in psychometric scales after excluding items i-1 and i-2, reducing the reliability of measurements. The exclusion of item i-5 (self-medication behaviour) reduced Cronbach’s alpha values of LS and LDS, confirming the usefulness of the LS to characterize behaviour of self-medication (Table 4).

**Table 4:** **Reliability of five-item structured self-report in dichotomous and Likert scales**

Parameters	Scales	Item				
		(i-1)	(i-2)	(i-3)	(i-4)	(i-5)
Item-to-total correlation	Dichotomous	0.59	0.55	0.30	0.09	−0.07
	Likert	0.72	0.58	0.38	0.26	0.51
	Likert dichotomized	0.70	0.61	0.26	0.20	0.35
	Overall dichotomous	0.65	0.58	0.28	0.15	0.18
Cronbach’s alpha when item was excluded	Dichotomous (0.508)*	0.22	0.24	0.43	0.58	0.60
	Likert (0.717)*	0.55	0.62	0.71	0.75	0.67
	Likert dichotomized (0.657)*	0.46	0.50	0.66	0.7	0.63
	Overall dichotomous (0.599)*	0.37	0.40	0.58	0.65	0.62

*Cronbach’s alpha original values.

The Spearman rank order correlation coefficient between pill count and psychometric measures ranged from 0.69 to 0.94, indicating a significant correlation between these variables, with higher values in LS and LDS. In fact, a strong correlation was seen between pill count and LS (Table 5). There was a good agreement between data from psychometric measures with pill count. Kappa values ranged from 0.683 to 0.936, with the high value in LS (Table 6).

**Table 5:** **Concurrent validation between pill count and psychometric measures in several scales**

Measure of adherence to treatment	Dichotomous	Likert	Likert dichotomized	Overall dichotomous
Dichotomous (n = 66)	-	-	-	-
Likert (n = 69)	−0.01	-	-	-
Likert dichotomized (n = 69)	−0.09	0.90*	-	-
Overall dichotomous (n = 135)	−0.10	0.43*	0.47*	-
Pill count (n = 135)	0.69*	0.94*	0.84*	0.77*

*Statistically significant.

**Table 6:** **Concordance of psychometric measures with pills count**

Scales	Pill count (PC)				Total	***Kappa*** ^***1***^	
	Non-adherence		Adherence				***C.O*** ^***2***^
	n	(%)	n	(%)			(%)
Dichotomous						0.683	
*Non-adherence*	13	*72.2*	5	*27.8*	18		87.9
*Adherence*	3	*6.3*	45	*93.8*	48		
Total	16	*24.2*	50	*75.8*	66		
Likert						0.936	
*Non-adherence*	23	*92.0*	2	*8.0*	25		97.1
*Adherence*	0	*-*	44	*100.0*	44		
Total	23	*33.3*	46	*66.7*	69		
Likert dichotomized						0.839	
*Non-adherence*	21	*87.5*	3	*12.5*	24		92.7
*Adherence*	2	*4.4*	43	*95.6*	45		
Total	23	*33.3*	46	*66.7*	69		
Overall dichotomous						0.771	
*Non-adherence*	34	*81.0*	8	*19.0*	42		90.4
*Adherence*	5	*5.4*	88	*94.6*	93		
Total	39	*28.9*	96	*71.1*	135		

^1^Values of Kappa according Rosner (2006). ^2^Concordance observed.

Accuracy and the capacity of five-item-self reported, to distinguish non-adherence in a group of non-adherent patients, as well the adherence in a group of probably adherent patients were high in psychometric measures (Table 7). Again, the high values were observed in LS. Finally, the application of five-item-self reported with responses in LS to patients with malaria by *P. vivax* indicates a non-adherence of 33.3% to malaria therapy. The main reason to low adhesion in psychometric measures were the miss taking the pills when the patients are feeling better or sick.

**Table 7:** **Sensitivity, specificity and accuracy of psychometric measures in several scales**

Measure of adherence to treatment	Sensitivity (%)	Specificity (%)	Accuracy (%)
Dichotomous (n = 66)	81.3	90.0	87.9
Likert (n = 69)	100	95.7	97.1
Likert dichotomized (n = 69)	91.3	93.5	92.8
Overall dichotomous (n = 135)	87.2	91.7	90.4

## Discussion

The adherence to malarial therapy is a challenge in endemic areas of seasonal transmission, such as Amazonia region, with several epidemiological characteristics associated with environmental and behavioural variables of human host, Plasmodium and mosquitoes [28, 29]. Malaria is an acute infectious disease with short treatment period, but the complexity of dosing regimens and the poverty in the region hamper the adherence with therapy, leading to high levels of low adherence or even non-adherence [30].

No single measure can be considered as gold standard for all types of adherence studies. The uses of biomarkers or the routine analysis of anti-malarials in biological media is not feasible in several endemic areas, and the uses of drug blood levels as a measure of adherence is complicated by the potential pharmacokinetics variability of these drugs. On other hand, self-report can be affected by different biases, since the patient tends to give the expected answer. In fact, the methods of self-report underestimate non-adherence, compared to pill count or biological assays. However, when properly structured and implemented self-report can provide valuable information on the behaviour of adherence.

To improve the reliability of the five-item-self-reported questionnaire the responses were compared among psychometric scales. As expected, LS showed the highest variation in response, because the greatest number of response options usually involves a greater variance and the information supplied will be better the higher the variance of the responses. The non-adherence to treatment was significantly lower when measured by psychometric scales, and by pill count. However, when the non-adherence was compared among measures of adherence to treatment the results were similar, with the highest values on the LS, corroborating the item response theory, in which the greatest number and variability of scores increases the likelihood of differentiating subjects, as well as high correlations with the various items [31, 32].

In this study the four-item-self reported originally proposed by Morisky *et al.* was modified with the inclusion of a fifth-item regarding self-medication, since a high numbers of treatments are taken at home without medical supervision. The inclusion of the fifth item did not modify the internal consistency of the questionnaire assessed by Cronbach’s alpha. The internal consistency was considered moderate when assessed in dichotomous scales and acceptable by LS [33, 34]. In addition, the exclusion of the fifth item decreased the internal consistency of LS and LDS, but increased in DS and ODS, indicating that LS, even after dichotomizing to LDS, increases the reliability of evaluating self-medication. The inclusion of the fifth item also increased the coefficient of correlation only on the LS.

The concordance between psychometric measures with pill count to estimate both adherence and non-adherence was high. Responses on LS showed a better concordance, which was reduced after its dichotomization to LDS. Dichotomous scales have several shortcomings when compared to LS, because they generally fall in sensibility and accuracy, and can generate false positive results underestimating the non-adherence. As an example, in LS a high frequency of answers “sometimes” in both items that report the deliberate interruption of treatment, which usually is not readily taken over by the patient, indicates probably, cognitive imprecision and, the interpretation of the answer “sometimes” on a dichotomous scale is highly subjective. Corroborating this finding, LS also presented higher values of sensitivity, specificity and accuracy when compared to dichotomous scales.

Pill count can be a feasible alternative to shortcomings of determining anti-malarials blood levels in endemic areas and was used to concurrent validation. Correlations of psychometric measures with pill count were considered excellent, with higher value in LS [35]. The main requirements to psychometric measures methods are: avoid simplistic dichotomies, be relatively inexpensive and easy to use and analyse, provide reliable, and provide a continuous record of compliance history. Thus, the results of this study indicate that the psychometric measures using structured questionnaires applied correctly are reliable for estimating adherence to malaria therapy. Answers on LS provide more valuables information on the behaviour of uses of anti-malarials. When applied to patients with *P. vivax* from Amazon basin, the five-item-self-reported questionnaire with response on LS estimate a non-adherence of 33.3%, which corroborate previous reports from others endemic areas [36, 37]. The interruption of treatment, both feel better or worse, was considered the most important reason to non-adherence.

## Conclusion

The five-item-self-reported questionnaire derived from the original Morisky questionnaire, with the addition of an item regarding self-medication behaviour with response on Likert scale is a feasible option for reliable estimation of adherence to malaria therapy in endemic areas.
